# 2-Styrylchromones Prevent IL-1β-Induced Pro-Inflammatory Activation of Fibroblast-like Synoviocytes while Increasing COX-2 Expression †

**DOI:** 10.3390/pharmaceutics15030780

**Published:** 2023-02-26

**Authors:** Ana Teresa Rufino, Mariana Lucas, Artur M. S. Silva, Daniela Ribeiro, Eduarda Fernandes

**Affiliations:** 1 LAQV, REQUIMTE, Laboratory of Applied Chemistry, Department of Chemical Sciences, Faculty of Pharmacy, University of Porto, Rua de Jorge Viterbo Ferreira No. 228, 4050-313 Porto, Portugal; 2 LAQV, REQUIMTE, Department of Chemistry, University of Aveiro, Campus Universitário de Santiago, 3810-193 Aveiro, Portugal; 3 Faculty of Agrarian Sciences and Environment, University of the Azores, Rua Capitão João d’Ávila—Pico da Urze, 9700-042 Angra do Heroísmo, Portugal

**Keywords:** 2-Styrylchromones, rheumatoid arthritis, fibroblast-like synoviocytes, inflammation, NF-kB, COX-2

## Abstract

Rheumatoid arthritis (RA) is characterized by systemic immune and chronic inflammatory features, leading to the destruction of the joints. Presently, there are no effective drugs able to control synovitis and catabolism in the process of RA. 2-Styrylchromones (2-SC) are a small group of compounds characterized by the attachment of a styryl group to the chromone core that have already been associated to a wide range of biological activities, including antioxidant and anti-inflammatory activities. The present study investigated the effect of a set of six 2-SC on the interleukin-1β (IL-1β)-induced increase of nitric oxide (^•^NO), inducible form of nitric oxide synthase (iNOS), cyclooxygenase-2 (COX-2), and matrix metalloproteinase-3 (MMP-3) expression levels in human fibroblast-like synoviocytes (HFLS), pointing to the role of nuclear factor kappa-light-chain-enhancer of activated B cells (NF-κB) activation in the process. From a set of six 2-SC, presenting hydroxy and methoxy substituents, the one presenting two methoxy substituents at C-5 and C-7 of A ring and a catechol group on B ring, significantly reduced ^•^NO production and the expression of its inducible synthase (iNOS). It also significantly reduced the catabolic MMP-3 protein expression. This 2-SC inhibited the NF-κB pathway by reversing the IL-1β - induced levels of cytoplasmatic NF-kB inhibitor alpha (IκBα), and decreasing the p65 nuclear levels, suggesting the involvement of these pathways in the observed effects. The same 2-SC significantly increased the COX-2 expression, which may indicate a negative feedback loop mechanism of action. The properties of 2-SC may be of great value in the development of new therapies with improved efficacy and selectivity towards RA, and thus deserve further exploitation and evaluation to disclose the full potential of 2-SC.

## 1. Introduction

Rheumatoid arthritis (RA) is an autoimmune disease characterized by progressive and chronic synovial inflammation, leading to irreversible cartilage and bone damage. It primarily affects the synovial membrane of the moving joints (hands, wrists, feet, knees, ankles, and elbows), but also involves the adjacent tissues, such as bone, muscle, and tendons [1,2,3]. Its effects include pain, swelling, stiffness, and in the end loss of joint function [1,3]. With a prevalence that varies between 0.3% and 1% in developed countries [3,4], World Health Organization (WHO) considers RA as one of the most prevalent chronic inflammatory diseases [5].

Fibroblast-like synoviocytes (FLS) represent a specialized cell type located in the synovium. They are recognized as the key players on the initiation and progression of RA and the major ones responsible for the destructive process of the joints [6,7]. Once activated, they produce their own inflammatory mediators, including cytokines, prostaglandins (PG), leukotrienes, nitric oxide (^•^NO), and large amounts of matrix metalloproteinases (MMP)-3, -9, and -13 in a process that leads to synovial inflammation perpetuation and to the cartilage and bone destruction observed in RA [6,7]. Moreover, inflammatory cytokines activate intracellular signaling pathways in the synovial membrane, including nuclear factor kappa-light-chain-enhancer of activated B cells (NF-κB), which assume a significant role in the process of synovial inflammation [8,9,10] by contributing to the activation/expression of several other inflammatory and immunomodulatory mediators, such as cyclooxygenase-2 (COX-2) and inducible nitric oxide synthase (iNOS). The activation of NF-κB is observed in the synovium of RA patients and arthritis animal models and the inhibition of the NF-κB signaling pathway has been shown to suppress the inflammatory response and joint destruction of collagen-induced arthritis in mice [11].

In the last decade, there was an expansion of the available RA therapeutic options whose application depends on the RA grade, duration, pre-existence of comorbidities, and response to the treatment, including glucocorticoids (e.g., prednisolone), conventional synthetic disease-modifying antirheumatic drugs (DMARDs) (e.g., methotrexate, hydroxychloroquine, and sulfasalazine), biological targeted DMARDs (bDMARD) (as tumor necrosis factor (TNF-α) inhibitors (e.g., etanercept, adalimumab, and infliximab), interleukin (IL)-6 receptor inhibitors (e.g., tocilizumab and sarilumab), and immunomodulators (e.g., abatacept and rituximab), synthetic targeted DMARDs (janus kinase (JAK) inhibitors (e.g., tofacitinib and baricitinib)), or a combination of the different drugs [12].

Despite the extensive research and the emergence of new therapeutic approaches and new drugs, there are still significant unwanted side effects associated with the DMARDs, including hepatotoxicity, cytopenia, and transaminase elevation for conventional DMARDs and gastrointestinal effects, lymphopenia, neutropenia, elevated cholesterol, and more infections susceptibility for JAK inhibitors, which demands a strict laboratory monitoring of the therapeutics in use [12,13]. Moreover, there is still a vast number of patients that do not respond positively to the existing therapeutic strategies [13]. So, new, safer, and more effective therapies, able to halt the disease progression and reverse the pathological process, are urgently required.

The synovial inflammation, a hallmark of RA, and the role of FLS on their initiation and progression make these cells and their chronic inflammatory and catabolic pathways expose a plethora of natural targets for the search of new effective molecules to stop or halt the progression of the disease.

**Figure 1:**
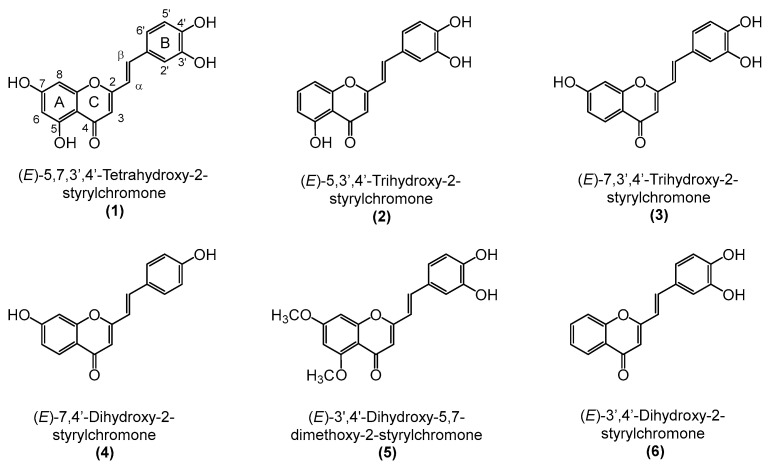
Chemical structures of the tested 2-styrylchromones (2-SC) (**1**–**6**).

2-Styrylchromones (2-SC), also known as 2-styryl-4*H*-chromen-4-one, constitute a small group of heterocyclic compounds, that contain a γ-benzopyrone ring with a styryl moiety at C-2 (Figure 1). These compounds, whose origin is mostly synthetic, have a recognized wide range of biological properties, namely antioxidant, antiviral, antifungal, antiallergic, antibacterial, antitumoral, and anti-inflammatory [14,15]. The already explored anti-inflammatory activities of 2-SC showed potential for reactive pro-oxidant species scavenging [16], modulation of human neutrophils’ oxidative burst [17], inhibition of NF-κB activation and production of cytokines/chemokine in THP-1 monocytes, inhibition of COX-1 activity, and leukotriene B4 production in human polymorphonuclear leukocytes [18,19], as well as inhibition of lymphocyte function-associated molecule-1/intercellular cell adhesion molecule-1-mediated cell adhesion, in HL-60 cells [20]. However, to the best of our knowledge, the anti-inflammatory effects of 2-SC in the context of RA and particularly on FLS have not been explored yet.

In this study, we evaluated the anti-rheumatoid arthritis effects of a set of 2-SC (Figure 1) presenting hydroxy (-OH) and methoxy (-OCH_3_) substituents by studying their role in IL-1β-treated human fibroblast-like synoviocytes (HFLS) cells and examining their impact on the protein expression of several inflammatory and catabolic events crucial for RA development.

## 2. Materials and Methods

### 2.1. Materials

HFLS growth medium, and HFLS growth medium without fetal bovine serum (FBS) were purchased from Cell Applications Inc. (San Diego, CA, USA). Recombinant Human IL-1β was obtained from PeproTech, (London, UK). Cell Counting Kit-8 (CCK-8), sodium nitrite (NaNO_2_), anti-β-actin antibody, and anti-rabbit HRP conjugated secondary antibody were obtained from Sigma-Aldrich (Burlington, MA, USA). COX-2, iKBα, and p65 antibodies were obtained from Cell Signaling (Danvers, MA, USA). MMP-3 and anti-mouse HRP conjugated secondary antibodies were obtained from Santa Cruz Biotechnologies (Santa Cruz, CA, USA), and iNOS antibody was obtained from Abcam (Cambridge, UK). Dimethylsulfoxide (DMSO) was obtained from Fisher Chemical™ (Loughborough, Leics, UK). All the 2-SC used in the work were synthesized as previously described [21].

### 2.2. Cell Culture and Treatment

HFLS were cultured in HFLS growth medium in a humidified incubator with 5% CO_2_ and 37 °C and 2–15 passages were used for experiments.

Before each experiment, HFLS were serum-starved, using HFLS growth medium without FBS, for at least 18 h and thereafter maintained in FBS-free medium. The 2-SC under study, diluted in DMSO at a concentration of 10 mM, were added to the HFLS cultures 30 min before the pro-inflammatory stimulus (IL-1β, 10 ng/mL) and maintained for the rest of the experimental period. The final concentrations of 2-SC, achieved in the culture medium, were indicated in the figures and their legends.

The final concentration of DMSO did not exceed 0.1% (*v*/*v*).

For signaling studies, the duration of the treatment was 30 min, and to evaluate the ^•^NO production and the protein expression of iNOS, MMP-3, and COX-2, the treatments lasted for 24 h.

### 2.3. Cell Viability Assay

Cell viability was determined by CCK-8 assay, which is a colorimetric assay for the determination of the number of viable cells based on the bioreduction of the WST-8 [2-(2-methoxy-4-nitrophenyl)-3-(4-nitrophenyl)-5-(2,4-disulphophenyl)-2*H*-tetrazolium, monosodium salt], into a water-soluble formazan dye in the presence of an electron carrier, 1-methoxy PMS. Briefly, HFLS cells (5 × 10^4^ cells/well) were seeded into 96-well plates and treated with 2-SC (0–50 μM) for 24 h. Two hours prior to termination, 10 μL of CCK-8 reagent was added to each well and further incubated at 37 °C. At the end of incubation, the optical density values at 450 nm were read by an automatic microplate reader (BioTek, Winooski, VT, USA).

### 2.4. Nitrite Assay

Nitrite measurement was determined based on the colorimetric detection of the reaction product of nitrite with naphthylethylenediamine dihydrochloride according to the Griess method, as previously described by Green et al. [22]. The concentration of nitrite, which reflects ^•^NO production, was measured in the cell-free supernatants collected from HFLS after treatment with 2-SC and/or IL-1β for 24 h (5 × 10^5^ cells/well). After the incubation period, 150 μL of culture medium was collected and placed in a 96-well plate, where were added to an equal volume of Griess reagent (0.2% naphthylethylenediamine dihydrochloride, and 2% sulphanilamide in 5% phosphoric acid) and the mixture was incubated for 30 min, in the dark at room temperature. The absorbance was measured at 570 nm using an automatic microplate reader. The absorbance of different concentrations of NaNO_2_ (0.5–50 μM) solution at 540 nm was measured to prepare a ^•^NO standard curve.

### 2.5. Western Blot Analysis

For Western blot analysis, 1 × 10^6^ HFLS were seeded in 6-well plates and treated with 2-SC for 24 h. At the end of the time allotted for the assay, total, cytoplasmatic, and nuclear cell extracts were prepared using lysis buffers, supplemented with proteases and phosphatases inhibitors. The protein concentration was determined by the Bradford method and cell lysates were denatured in sample buffer (0.5 M Tris-HCl, pH = 6.8, 2% (*v*/*v*) sodium dodecyl sulphate, 10% glycerol, 25% (*v*/*v*) 2-mercaptoethanol and bromophenol blue). Proteins (25 μg/well) were separated by SDS-PAGE and transferred to a polyvinylidene fluoride (PVDF) membrane. The membrane was blocked in Tris-buffered saline with 0.1% Tween solution and was supplemented with 5.0% skim milk powder for 1 h at room temperature and subsequently probed, overnight, with the following primary antibodies: mouse monoclonal anti-human iNOS; mouse polyclonal anti-human IκBα rabbit monoclonal anti p65; rabbit monoclonal anti-COX-2; and mouse monoclonal anti-human MMP-3. Mouse anti-actin monoclonal antibody was used to detect actin as the loading control. Anti-mouse and anti-rabbit HRP-conjugated secondary antibodies were used as secondary antibodies. Immune complexes were detected with the Clarity™ western ECL substrate using Chemidoc Imaging System (Biorad, Hercules, CA, USA), and the bands were analyzed using QLICS software (version 7.0, Total Lab). The results were normalized by calculating the ratio between the intensities of the bands corresponding to the protein of interest and the actin used as the loading control.

### 2.6. Statistical Analysis

Results are presented as mean ± standard error of the mean (SEM) corresponding to at least 3 independent experiments. Statistical analysis was performed using GraphPad Prism (version 7.0). The statistical analysis was performed using the paired t-test for a comparison of each condition with its respective positive (IL-1β treated) or negative (untreated cells) controls. Results were considered statistically significant at *p* < 0.05.

## 3. Results

### 3.1. Evaluation of Cytotoxicity and Selection of Non-Cytotoxic Concentrations of the 2-SC

In order to determine the non-cytotoxic concentrations of 2-SC in the assay conditions, 2-SC (1), (3), (4), (5), and (6) were tested at concentrations up to 50 μM. The 2-SC (2) was only tested at concentrations up to 25 μM, according to non-published data from our group, indicating its increased cytotoxicity when compared to their counterparts.

**Figure 2:**
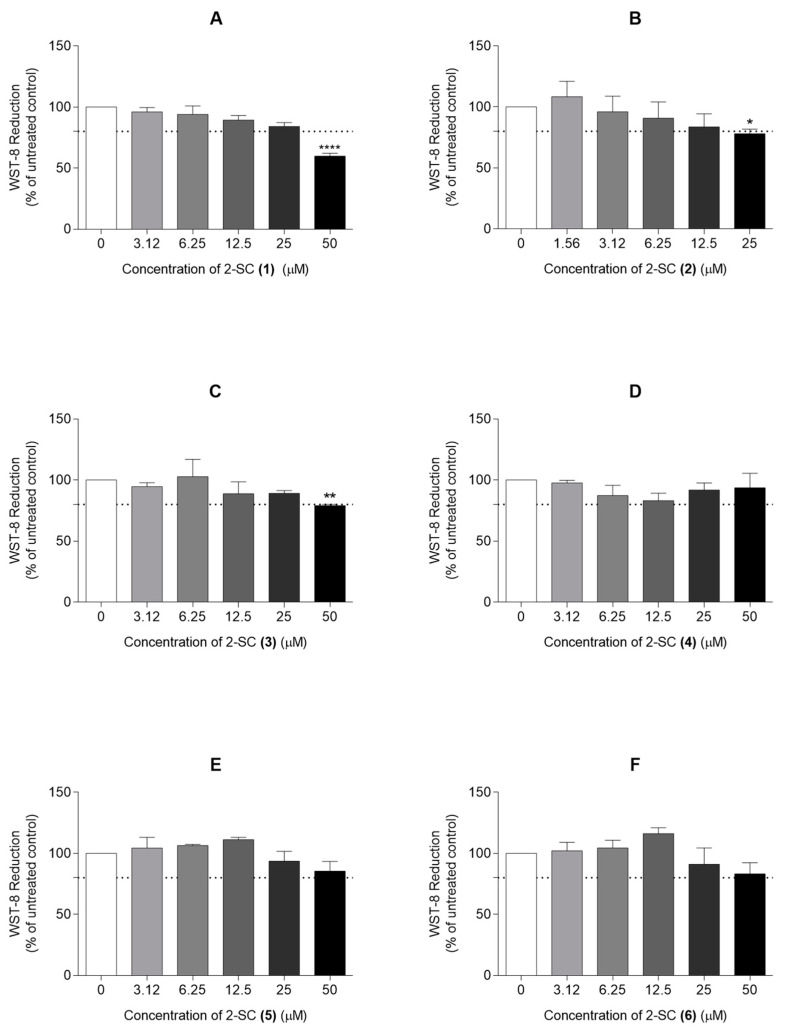
Effect of 2-Styrylchromones (2-SC) on the viability of human fibroblast-like synoviocytes (HFLS) (**A**–**F**). Cell viability was assessed in cell cultures treated with the indicated concentrations (0–50 μM) of the 2-SC (1 to 6) for 24 h. Each column represents, at least, 4 independent experiments. The dotted line represents 80% of the maximal absorbance below which cell viability is considered compromised. * *p* < 0.05, ** *p* < 0.01 and **** *p* < 0.0001 relative to the respective control (untreated) cells.

Figure 2 shows that the exposure of HFLS for 24 h to a 50 μM concentration of 2-SC (1) and (3) and 25 μM of 2-SC (2) decreased cell viability by more than 20%. On the contrary, the 2-SC (4), (5), and (6) were completely devoid of significant cytotoxicity at concentrations up to 50 μM. Therefore, subsequent experiments were performed using the identified non-cytotoxic concentrations for each 2-SC, namely 12.5 μM for 2-SC (2), 25 μM for 2-SC (1) and (3), and 50 μM for 2-SC (4), (5), and (6).

### 3.2. Effects of 2-SC on IL-1β-Induced Nitric Oxide Production and iNOS Protein Expression

Several documents expose that, in RA, ^•^NO is implicated in most of the pathogenic processes, including inflammation, angiogenesis, and ultimately in tissue destruction. The iNOS has been described as responsible for the overproduction of ^•^NO in the synovial joints affected by RA. Several cells of the joint including FLS can be induced by pro-inflammatory cytokines to produce ^•^NO in vitro.

Regarding the importance of ^•^NO and iNOS in the development and maintenance of the inflammatory process in RA, we investigated the role of 2-SC in the modulation of these two parameters. Furthermore, we begin by understanding the appropriateness of the method chosen for this evaluation, the reason we investigated the effect of IL-1β relative to control values on the ^•^NO production and iNOS protein expression.

**Figure 3:**
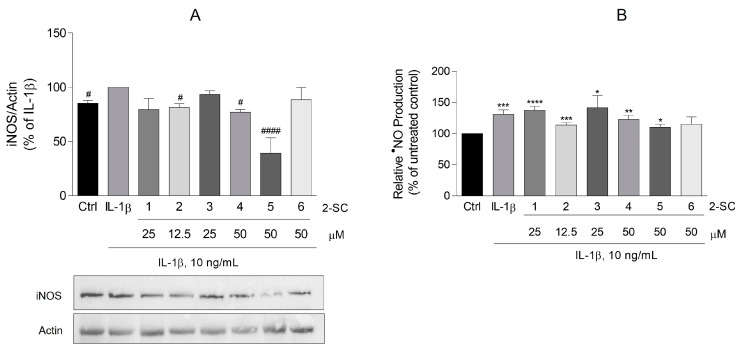
Effect of 2-Styrylchromones (2-SC) on IL-1β-induced inducible nitric oxide synthase (iNOS) protein expression (**A**) and nitric oxide (^•^NO) production (**B**) in human fibroblast-like synoviocytes (HFLS). Cell cultures were left untreated (control, Ctrl) or treated with IL-1β (10 ng/mL), for 24 h, following pre-treatment for 30 min with the indicated concentrations of the 2-SC. Each column represents the mean ± SEM of, at least, 3 independent experiments. * *p* < 0.05, ** *p* < 0.01, *** *p* < 0.001, **** *p* < 0.0001 relative to Ctrl. ^#^ *p* < 0.05, ^####^ *p* < 0.0001 relative to IL-1β treated cells.

Thus, treatment of HFLS with 10 ng/mL of IL-1β for 24 h significantly induced the expression of iNOS (Figure 3A). Accordingly, HFLS stimulated with IL-1β also increased the ^•^NO production by around 30% relative to the control cells, as revealed by the concentration of nitrite accumulated in the culture medium (Figure 3B).

Of the 2-SC tested, (5), in a concentration of 50 μM, elicited an expressive decrease of the iNOS expression (61 ± 14%) (Figure 3A), which surprisingly is not accompanied by a similar reduction of ^•^NO production, since this decrease stays at 20 ± 6% relative to the IL-1β treated cells. However, even at a modest but statistically significant level, 2-SC (2) (12.5 μM) and (4) (50 μM) also reduced the levels of both iNOS expression (19 ± 3% and 23 ± 3%, respectively) (Figure 3A) and ^•^NO production (17 ± 6 and 8 ± 4%, respectively) (Figure 3B), when compared to the IL-1β treated cells.

### 3.3. Effect of 2-SC on IL-1β-Induced NF-kB Activation

**Figure 4:**
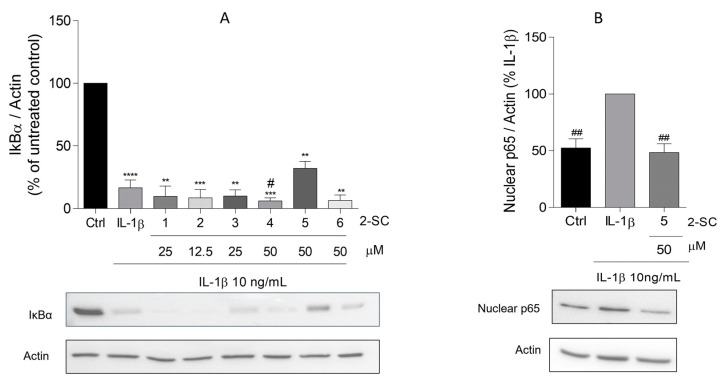
Effect of 2-Styrylchromones (2-SC) on IL-1β-induced NF-κB activation, evaluated as the levels of total IκB-α (**A**) and nuclear p65 (**B**) on human fibroblast-like synoviocytes (HFLS) cells that were left untreated (control, Ctrl) or treated with IL-1β (10 ng/mL), for 30 min, following pre-treatment for 30 min with the indicated concentrations of the 2-SC. Each column represents the mean ± SEM of 3 independent experiments. ** *p* < 0.01, *** *p* < 0.001, **** *p* < 0.0001 relative to Ctrl. ^#^ *p* < 0.05, ^##^ *p* < 0.01 relative to IL-1β treated cells.

The activation of NF-κB is observed in the synovium of RA patients and arthritis animal models and the inhibition of the NF-κB signaling pathway has been shown to suppress the inflammatory response and joint destruction of collagen-induced arthritis in mice. Moreover, available data show that the activation of inflammatory and catabolic genes, including iNOS, COX-2, and MMP-3, are mostly regulated by the NF-κB transcription factor and the activation of NF-κB is dependent on the phosphorylation and degradation of IκBα, its natural inhibitor, and the translocation of its p65 subunit to the nucleus [23]. In this sense, to determine whether 2-SC inhibits the activation of NF-κB in IL-1β stimulated HFLS, the protein levels of total IκBα and the p65 nuclear levels were evaluated by Western blot. The obtained results showed that a 30 min treatment with IL-1β significantly decreased the total form of IκBα, being barely undetectable when compared to the non-treated control cells (Figure 4A). Our results also show that the IL-1β-induced reduction of IκBα levels (17 ± 6%) was slightly reversed by the 2-SC (5) (32 ± 6%) in cells pre-treated with 50 μM of the 2-SC. Accordingly, in the same conditions, the 2-SC (5) significantly reduced the IL-1β induced p65 nuclear levels (52 ± 8%) (Figure 4B).

### 3.4. Effect of 2-SC on IL-1β-Induced COX-2 and MMP-3 Expression

The degradative process observed in RA occurs in part due to the chronic and persistent inflammatory process in joint tissues, and this is ultimately due to a significant increase in catabolic components such as MMPs. Thus, finding mechanisms/and or molecules that control not only the inflammatory process, but also the degradative process that occurs throughout the joint may work as a new opportunity for the development of new therapies to alter the normal degradative course of the disease.

High levels of COX-2 and MMP-3 are present in RA synovial fluid of patients with RA when compared with the synovial fluid of normal subjects [24].

**Figure 5:**
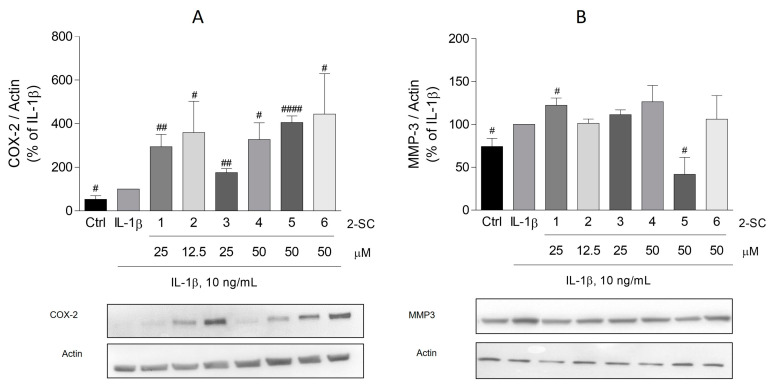
Effect of 2-Styrylchromones (2-SC) on IL-1β-induced cyclooxygenase (COX-2) (**A**) and metalloproteinase-3 (MMP-3) (**B**) protein expression in human fibroblast-like synoviocytes (HFLS). Cell cultures were left untreated (control, Ctrl) or treated with IL-1β (10 ng/mL), for 24 h following pre-treatment for 30 min with the indicated concentrations of the 2-SC. Each column represents the mean ± SEM of, at least, 3 independent experiments. ^#^ *p* < 0.05, ^##^ *p* < 0.01, ^####^ *p* < 0.0001 relative to IL-1β treated cells.

To determine whether 2-SC affects the IL-1β-induced production of COX-2 and MMP-3, their protein levels were evaluated by Western blot. As expected, treatment of HFLS with IL-1β for 24 h, significantly induced the expression of both COX-2 (Figure 5A) and MMP-3 (Figure 5B).

Regarding the effect of the 2-SC under study on COX-2, surprisingly, all of them, at the maximum concentration tested, significantly induced the expression of COX-2. This effect is especially relevant for the 2-SC (5) and (6), which elicited an expression approximately four-fold bigger than the cells treated with IL-1β alone (406 ± 30% and 445 ± 184%, respectively) (Figure 5A).

Contrarywise, of the 2-SC tested, only (5) produced a significant decreasing effect on IL-1β induced MMP-3 (Figure 5B). At a concentration of 50 μM, it elicited a reduction of MMP-3 to values even lower than those of untreated cells (42 ± 19%). On the other hand, 2-SC (1) slightly increased the MMP-3 levels (22 ± 9%) relative to the IL-1β-treated cells.

## 4. Discussion

RA is a systemic immune and inflammatory disease that leads to the destruction of the joint ligaments, cartilage, and bone. For the majority of patients, RA is progressive and shortens their life quality and expectancy [25]. Unfortunately, there are still many gaps regarding the pathophysiology of the disease and its treatment.

The challenge of finding new treatments is not only dependent on the discovery of new molecules, but also on the use of models that could balance the proximity to the disease features with the experimental feasibility and the reproducibility of the results [26]. Cell-based in vitro assays for the therapeutic evaluation of new molecules comprise the use of a variety of cytokine-treated isolated articular cells involved in the pathogenesis of the RA including FLS. For their cost-effectiveness, convenience, and easiness of use, and despite their limitations, the use of FLS is still wide [26,27]. The same approach can be made for the pro-inflammatory cytokine IL-1β. It is present in high levels in the synovial fluid and peripheral blood of patients with RA [10], and systemic levels of IL-1β correlate with disease activity. Most of this cytokine present in inflamed joints in RA is produced by macrophages and synoviocytes [28]. Thus, this cytokine is frequently used to induce in vitro inflammatory markers, characteristic of RA, including iNOS [29], an enzyme capable of generating high levels of ^•^NO, a radical which excess contributes to the pathogenesis of RA, and COX-2 [30]. The latter, by producing PGs, is frequently associated with synovial inflammation by increasing local blood flow and vasopermeability. These observations are corroborated in our cellular model, with an increased expression of iNOS, ^•^NO, and COX-2, elicited by IL-1β, validating the chosen model.

The inflammatory environment of the joint also leads to the aggressive feature of RA by promoting the degradation of the joint tissues, in a process that is mediated by matrix MMPs, a family of proteases capable of ‘digesting’ basement membranes and extracellular matrix proteins [31].

Therefore, the present study investigated the effect of a set of 2-SC on the IL-1β-induced increase of ^•^NO, iNOS, COX-2, and MMP-3 expression levels in HFLS, pointing to the role of NF-kB activation in the process. 2-SC have already demonstrated significant effects on the modulation of NF-kB, one of the most important transcription factors of the inflammatory pathway, in relevant cells of the immune system [18]. Nevertheless, the present results show clear differences in the ability of the tested 2-SC to inhibit relevant mediators of inflammation in the cell model of RA under use. To the best of our knowledge, this is the first study to provide evidence of the ability of 2-SC to regulate IL-1β-induced inflammatory and catabolic mediators in cultured HFLS.

The present results show that from the set of 2-SC tested, the 2-SC (5), which presents two methoxy (-OCH_3_) substituents at C-5 and C-7 of the A ring and a catechol group on the B ring, efficiently decreases the expression of iNOS induced by IL-1β. Surprisingly, the reduction of iNOS by this 2-SC was not completely echoed with a reduction of ^•^NO production. Sousa et al. [32] synthesized a series of new 3-hydroxy-2-SC and studied their ability to scavenge ^•^NO. The IC50 found for the methoxylated in the A ring was slightly higher than the IC50 found for the hydroxylated 2-SC [32]. Interestingly, the presence of methoxy groups in the scaffold of flavonoids, the synthetic precursors of 2-SC, even in the presence of hydroxy groups on the B ring, is indeed described as decreasing their anti-nitrosative activity [33]. The observed decrease in ^•^NO elicited by the other 2-SC could be due to their capacity to act as scavengers of this radical as it has already been demonstrated by Gomes et al. [16]. Moreover, even though the literature shows that the majority of human cells, and particularly synoviocytes, were capable of producing high-output levels of ^•^NO [34], in vitro, it also shows that human normal cells appear to be more resistant to ^•^NO-mediated effects, which may be due to a more effective and higher capacity to call protective mechanisms [29,35].

Screening of molecules presenting possible therapeutic efficiency towards the inhibition of MMPs has been considered an interesting target to be explored in the RA context [36,37,38,39]. Our results also showed that the 2-SC (5) was capable of significantly reducing IL-1β-induced MMP-3 expression. This MMP can degrade proteoglycan, collagens, fibronectin, gelatin, and laminin and it is considered to be especially important for both enzyme activity and for the full activation of other collagenases [39,40]. Therefore, the observed effect for the 2-SC (5) reveals an anti-protease potential, important to partially block the pathogenic function of synovial fibroblasts in RA.

Moreover, since the activation of several mediators of inflammatory and catabolic processes [41], including iNOS, COX-2, and MMPs genes [24,42] are intrinsically regulated by the NF-κB, the ability of 2-SC to inhibit IL-1β-induced NF-κB activation was also evaluated in order to further elucidate the mechanism of action behind the observed effects of the 2-SC (5). NF-κB activity is mediated by a family of transcription factor subunits that bind DNA as hetero- or homodimers. These NF-kB subunits (p65 and p50) are present in the cytoplasm in an inactive form, bound to inhibitory proteins, namely IκBα [23]. A variety of extracellular stimuli, including IL-1β, induce the phosphorylation of subsequent degradation of IκBα. The dissociation of NF-kB from cytoplasmic inhibitors is followed by the translocation of their subunits complex to the nucleus, followed by binding to specific DNA sequences responsible for the transcription of the relevant genes. p65 (Rel A) is the major component of NF-kB dimers found in HFLS [43]. The effects of 2-SC on NF-kB modulation have already been demonstrated by Gomes et al. [18]. The present results also show that the 2-SC (5) was capable of significantly reverse the NF-kB activation induced by IL-1β in HFLS, especially by the observation of the nuclear levels of p65. This suggests that the suppression of NF-kB activation by this 2- SC of iNOS and MMP-3 production is likely to be, at least partially, due to the direct modulation of NF-kB subunits binding to the promoter regions of the genes coding for these proteins. Interestingly the substitution pattern of the active 2-SCs is slightly different from that reported in previous studies. While the presence of -OH at the C-3′ and C-4′ positions of the B ring seemed to generically favor anti-inflammatory activity, in the present study, and despite appearing to be a favorable conjugation, as seen by the comparison between the 2-SC (3) and (4), the anti-inflammatory effects and particularly the effect through NF-kB is favored by the presence of methoxy groups on A ring. Finally, a surprising result also shows that the 2-SC (5) significantly increases the expression of COX-2. This effect seems to be contrary to the expectations regarding the canonical activation of COX-2 expression via NF-kB activation [44]. Nevertheless, even though long assumed as a pro-inflammatory mediator, COX-2 and the major products resulting from their activity, PGs, have already shown important anti-inflammatory activities. Following activation, COX-2 generates PGH_2_, which in turn serves as a substrate for many other reactions catalyzed by PG synthases. The levels of those synthases tend to decrease in the acute inflammation phase and increase in the resolution phase, suggesting their role in inflammation resolution [45]. In addition to this potential effect, other pieces of evidence arise showing that PGE_2_, the classic model of a pro-inflammatory mediator, also presents anti-inflammatory effects that can be both potent and context-dependent, a subject substantially explored by Scher et al. [45]. In brief, the literature shows that PGE_2_ analogs have been related to anti-inflammatory effects in an inflammatory arthritis animal model [46] and that PG_E1_ and PG_E2_ inhibited IL-1β and TNF-α-induced MMP-1 secretion in synovial fibroblasts, while non-selective and COX-2 selective inhibitors had the opposite effect [47]. Furthermore, studies on PGE_2_ over the NF-kB activity surprisingly show that COX-2-generated PGE_2_ inhibited the nuclear translocation of p65 [45,48,49,50] and inhibited NF-kB activation via the inhibition of IkkB, the kinase responsible for the phosphorylation of the IκB inhibitors [51]. All this suggests the existence of a negative feedback loop between the transcription factor and the COX-2. The existence of this feedback loop affecting NF-kB and COX-2 synthesis and action help to explain the results obtained using 2-SC (5) in this work and support their anti-inflammatory effect on HFLS.

## 5. Conclusions

In conclusion, from the six 2-SC tested, the only one presenting methoxy substituents was able to significantly decrease ^•^NO production, iNOS, and MMP-3 expression in a process at least partially mediated by NF-kB. Despite the available literature advocating that the presence of −OH at C-3′ and C-4′ on the Bring of 2-SC generically favors the anti-inflammatory activity [14], our study shows that, at least in the assay conditions, these substitutions are not enough to guarantee the biological effect. Nevertheless, its presence together with the methoxy substituents seems to be particularly relevant for the observed activities, since their absence (2-SC (6)) or substitution by hydroxyl groups (2-SC (1)) leads to a loss of function. Moreover, this same 2-SC (5) significantly increased the expression of COX-2, which may indicate a negative feedback loop mechanism of action. We are aware that some limitations of this study, including the ability of primary cells to grow and respond to the treatments performed, the diversity of mediators and processes involved in the RA disease that cannot be replicated with the model used, and the limited number of 2-SC tested, can limit and bias the obtained conclusions. Despite the limitations of the method and the evident need to further explore the molecular mechanisms involved, to our knowledge, the literature does not show any other 2-SC or similar polyphenolic structure presenting the effect described for 2-SC (5) on the modulation of NF-kB and COX-2. Nevertheless, it is particularly interesting for future studies of structure-activity relationships and further description of mechanisms of action. Finally, these properties may be of great value in the development of new 2-SC with improved efficacy and selectivity towards RA, which should be further explored and carefully evaluated to reveal the full potential of 2-SC on the RA treatment.
